# A retrospective prognostic evaluation analysis using the 8th edition of the American Joint Committee on Cancer staging system for breast cancer

**DOI:** 10.1007/s10549-018-4682-5

**Published:** 2018-01-31

**Authors:** Sae Byul Lee, Guiyun Sohn, Jisun Kim, Il Yong Chung, Jong Won Lee, Hee Jeong Kim, Beom Seok Ko, Byung Ho Son, Sei-Hyun Ahn

**Affiliations:** Division of Breast Surgery, Department of Surgery, University of Ulsan College of Medicine, Asan Medical Center, 88, Olympic-ro 43-gil, Songpa-Gu, Seoul, 05505 Korea

**Keywords:** AJCC cancer staging, Biomarker, Breast cancer, Subtype, Prognostic factors

## Abstract

**Purpose:**

Breast cancer is a group of diseases with different intrinsic molecular subtypes. However, anatomic staging alone is insufficient to determine prognosis. The present study analyzed the prognostic value of the American Joint Committee for Cancer (AJCC) 8th edition cancer staging system.

**Methods:**

This retrospective, single-center study included breast cancer cases diagnosed from January 1999 to December 2008. We restaged patients based on the 8th edition AJCC cancer staging system and analyzed the prognostic value of the anatomic and prognostic staged groups. Follow-up data including disease-free survival (DFS), overall survival (OS), and clinic-pathological data were collected to analyze the differences between the two staging subgroups.

**Results:**

The study enrolled 7458 breast cancer patients with a 98.7-month median follow-up. Both the 5-year DFS and OS were significantly different between the anatomic and prognostic staged groups. The 5-year OS according to disease subtype was as follows: hormone receptor-positive/human epidermal growth factor receptor 2-negative [HR(+)/HER2(−)], 90.9%; HR(+)/HER2(+), 84.7%; HR(−)/HER2(+), 81.1%; and HR(−)/HER2(−), 80.9%. According to the anatomic stage, the 5-year OS of patients with stage III HR(+)/HER2(−) disease was superior to that of patients with stage II HR(−)/HER2(−) disease (88.3 vs. 86.5%). Per the prognostic stage, both the 5-year DFS and OS rates of patients with stage II HR(−)/HER2(−) disease were higher than those of patients with stage III HR(+)/HER2(−) disease (90.1 and 94.3% vs. 79.1 and 88.9%).

**Conclusions:**

The prognostic staging system is a refined version of the anatomic staging system and encourages a more personalized approach to breast cancer treatment.

## Introduction

Like in other types of cancer, staging is the important step in the treatment of breast cancer. Thus, standardized staging tools are needed. The American Joint Committee for Cancer (AJCC) staging system is a widely applied tool used by physicians to predict disease progression and make therapeutic decisions [1]. The first edition of the AJCC was published in 1977, in which the TNM (primary tumor [T], regional lymph nodes [N], and distant metastasis [M]) system was reported [2]; the newest edition, the 8th edition, was published in October 2016, which will be implemented in January 2018 [3].

In 2011, attendees of the 12th St. Gallen Consensus Meeting [4] suggested that breast cancer should be divided into four subtypes: luminal A [estrogen receptor (ER) positive and/or progesterone receptor (PR) positive, human epidermal growth factor receptor 2 [HER-2] negative], luminal B (ER positive and/or PR positive, HER-2 positive), HER-2 positive (ER negative, PR negative, HER-2 positive), and triple negative (ER negative, PR negative, HER-2 negative) [5, 6]. Currently, breast cancer is generally recognized as a heterogeneous disease with a variety of clinical, pathologic, and molecular characteristics [5–7]. Thus, it can be said that breast cancer is a heterogeneous disease composed of distinct biological subtypes with various therapeutic responses and results [8]. These differences can be caused by genetic influences, lifestyle, nutritional diversity, and environmental factors [5].

Thus, the prognosis of breast cancer is known to be affected not only by the TNM stage but also by the subtype. The identification of these subtypes in the treatment of breast cancer has led to a change from standardized therapy to tailored therapy [9]. Therefore, the 8th edition of the AJCC staging system features four new biological factors: tumor grade, ER and PR expression, HER-2 expression, and multigene panels [3]. However, the new staging system needs to be further validated in terms of the clinical utility of tumor subtype, clinical setting, locality and population, and patient long-term benefits.

In this retrospective study, we analyzed the clinical significance of the prognostic staging system proposed in the 8th edition of the AJCC cancer staging system for breast cancer. The objective of the present study was to evaluate the validity of the 8th edition AJCC staging system for breast cancer based on data collected from the Asan Medical Center database.

## Methods

### Patients and clinical data

We reviewed the data for 11,116 patients with breast cancer who were treated at Asan Medical Center between January 1999 and December 2008. The median follow-up period for the entire cohort was 98.7 months (range 0–269.5 months). Patients with missing data regarding ER (*n* = 295), PR (*n* = 301), and HER-2 (*n* = 493) status were excluded as we were unable to classify these cases as luminal, HER-2 overexpressing, or triple negative. A total of 2490 patients with missing information regarding histologic grade were excluded. Women diagnosed with breast carcinoma in situ (*n* = 891) were also excluded. Finally, 7458 cases of invasive breast cancer were eligible for analysis. All of the patients’ information and tumor characteristics were retrieved from our retrospectively collected database. The database included information such as age, clinical manifestations, clinical and pathologic staging according to pathologic data, surgical methods, types of adjuvant treatment modalities, type of recurrence, and follow-up period.

We restaged all registered patients using the AJCC 8th anatomical and prognostic staging system [3]. The staging of cancer on the basis of the T, N, and M categories is considered an anatomic staging system. The prognosis staging system was based on breast cancer patients who were provided appropriate endocrine and/or systemic chemotherapy, as well as on the anatomical T, N, and M stage, tumor grade, biomarker status (ER, PR, and HER-2). This study was reviewed and approved by the Institutional Review Board of Asan Medical Center (20150185).

### Pathological data

Pathological data, including tumor size, number of axillary lymph node metastases, ER status, PR status, and HER-2 status were evaluated at the Department of Pathology in Asan Medical Center. The method for determining ER and PR status varied during the study period. ER and PR status have been determined immunohistochemically since 2000. Before 2000, ER and PR status were evaluated using the dextran charcoal-coating method or via an enzyme immunoassay. ER and PR expression were evaluated on the basis of intensity (0 indicates negative; 1, weakly positive; 2, intermediately positive; and 3, strongly positive) and staining percentage (1 indicates < 10%; 2, 10% to one-third; 3, one-third to two-third; and 4, more than two-third). The immunoreactive score determined by totaling the intensity and percentage scores was divided into 4 groups [negative (0–1), weakly positive [2, 3], intermediately positive [4, 5], and strongly positive [6, 7]). Intermediately and strongly positive scores indicated positive expression. ER and PR expression were considered to be positive if more than 10% of cells showed positivity. However, indications for endocrine therapy were considered even in patients with less than 10% of positive cells.

HER2 status has been evaluated since 2000. Immunohistochemistry was performed in a BenchMark XT autostainer using the OptiView DAB Detection Kit for HER2 (cat. 800-4422, clone 4B5, dilution 1:8, Ventana Medical Systems, Tucson, AZ, USA). The results were graded according to the level of coloring of the cell membranes of the cancer cells. The cases wherein less than 10% of the tumors cells stained positively were graded as 0, cases wherein membrane staining was partial but occurred in greater than 10% of the tumor cells were scored as 1+ , cases wherein entire cell membranes stained modestly were graded as 2+ , and cases wherein the entire cell membranes stained strongly but occurred in greater than 30% of the tumor cells were graded 3+ . For the HER-2 overexpression analysis, cases graded 0, 1+ or 2+ were considered to be negative. Cases graded 2+ were evaluated via fluorescence in situ hybridization and cases graded 3+ were regarded as positive.

### Survival

The survival duration for each patient was determined as the time (in months) between the date of initial diagnosis until the date of death, date of loss to follow-up, or the closing date for follow-up. Disease-free survival (DFS) was defined as the time from surgery to the first appearance of initial relapse (locoregional or disseminated). Overall survival (OS) was defined as the time from surgery to the time of death.

### Statistical analysis

Other data analysis was performed using SPSS version 21.0 (SPSS Inc., Chicago, USA). A linear regression analysis and the Chi-square test were used to determine the differences in each parameter over time, and the means of continuous variables such as age among different groups were compared using the *t* test. Survival curves were generated using the Kaplan–Meier method, and the significance of survival differences among the selected variables was verified using the log-rank test. The univariate Cox regression analysis was used to estimate hazard ratios. The multivariate Cox regression analysis with the backward elimination method was used to estimate hazard ratios and to identify independent prognostic factors. All reported *p* values are two-sided, and a value below 0.05 was considered to indicate statistical significance.

## Results

The general characteristics of the patients analyzed are shown in Table 1. The mean age of the patients was 47.6 years, and 46.3 and 53.7% of patients underwent BCS and mastectomy, respectively; in terms of *T* stage, 53.8 and 40.5% of cases were classified as *T*1 and *T*2, respectively, and in terms of *N* stage, 57.3 and 28.2% were classified as *N*0 and *N*1, respectively. In terms of tumor subtype according to hormone receptor (HR) and HER2 status, HR(+)/HER2(−) was found to be the most common (52.9%); moreover, 58.8, 71.3, and 69.8% of patients received radiation therapy, chemotherapy, and antihormonal therapy, respectively (Table 1).

**Table 1:** Clinicopathologic characteristics of the 7458 enrolled patients

Factors	*N*	%
Age at diagnosis (years old)		
< 35	557	7.5
35–50	4464	59.9
> 50	2437	32.6
Operation methods		
BCS	3451	46.3
Mastectomy	4005	53.7
Unknown	2	0
*T* stage		
*T*0	1	0
*T*1	4013	53.8
*T*2	3019	40.5
*T*3	316	4.2
*T*4	109	1.5
*N* stage		
*N*0	4270	57.3
*N*1	2106	28.2
*N*1mi	168	2.3
*N*2	550	7.4
*N*3	364	4.8
Histologic grade		
*G*1	516	6.9
*G*2	4076	54.7
*G*3	2866	38.4
Nuclear grade		
*G*1	384	5.2
*G*2	3572	47.9
*G*3	2610	35.0
*G*X	3	0
Unknown	889	11.9
Lymphovascular invasion		
Negative	4437	59.5
Positive	1686	22.6
Unknown	1335	17.9
Estrogen receptor		
Negative	2921	39.2
Positive	4537	60.8
Progesterone receptor		
Negative	3496	46.9
Positive	3962	53.1
HER2(IHC)		
Negative	5476	73.4
Positive	1982	26.6
Subtype		
HR+/HER2−	3944	52.9
HR+/HER2+	970	13.0
HR−/HER2+	1012	13.6
HR−/HER2+	1532	20.5
Radiation therapy		
Yes	4386	58.8
No	3039	40.7
Unknown	33	0.5
Chemotherapy		
Yes	5316	71.3
No	2096	28.1
Unknown	46	0.6
Antihormonal therapy		
Yes	5202	69.8
No	2191	29.4
Unknown	65	0.8

*HER2* human epidermal growth factor receptor-2, *IHC* immunohistochemistry

Stage IA tumors were found to be the most common type (38.5%) in terms of anatomic stage, followed by stage IIA tumors (29.1%); however, in terms of prognostic stage, stage IB tumors were the most common (24.1%), followed by stage IIIA (16.4%) and stage IIB (14.0%) tumors. When the cases were analyzed according to anatomic stage, the proportion of patients with stage III (14%) disease was smaller than that of patients with stage I (39.9%) and stage II (45.4%) disease. However, when the cases were analyzed according to prognostic stage, the proportion of patients with stage III disease increased to 32.5%, possibly because these cases were staged as stage I and II according to the anatomic stage (Table 2). Herein, the subtype at stage I in the anatomic stage is the order HR(+)/HER2(−) (58.5%), HR(−)/HER2(−) (17.6%), and they were similar in stage II and III. By the way, in the prognostic stage, HR(+)/HER2(−) is the most common (67.3%), but no HR(−)/HER2(−) at stage I. In addition, the rate of HR(−)/HER2(−) in stage III was 22.5% in the anatomic stage, but increases to 41.5% in the prognostic stage (Table 3).

**Table 2:** The distribution according to stage by the 8th edition of the AJCC anatomic staging system and prognostic staging system

	Anatomic stage		Prognostic stage	
	Number	%	Number	%
IA	2870	38.5	667	8.9
IB	104	1.4	1797	24.1
IIA	2171	29.1	1029	13.8
IIB	1217	16.3	1043.	14.0
IIIA	654	8.8	1224	16.4
IIIB	78	1.0	264	3.5
IIIC	364	4.9	937	12.6
Anonymous	0	0	497	6.7
Total	7458		7458

**Table 3:** The distribution according to subtype by the 8th edition of the AJCC anatomic staging system and prognostic staging system

	Anatomic stage		Prognostic stage	
	Number	%	Number	%
I				
HR+/HER2−	1739	58.5	1658	67.3
HR+/HER2+	338	11.4	632	25.6
HR−/HER2+	372	12.5	174	7.1
HR−/HER2−	525	17.6	0	0
Total	2974	100.0	2464	100.0
II				
HR+/HER2−	1710	50.5	1137	54.9
HR+/HER2+	478	14.1	112	5.4
HR−/HER2+	441	13.0	297	14.3
HR−/HER2−	759	22.4	526	25.4
Total	3388	100.0	2072	100.0
III				
HR+/HER2−	495	45.2	826	34.1
HR+/HER2+	154	14.1	100	4.1
HR−/HER2+	199	18.2	493	20.3
HR−/HER2−	248	22.5	1006	41.5
Total	1096	100.0	6961	100.0
Anonymous	0	0	497	
Total	7458		7458	

**Fig. 1:**
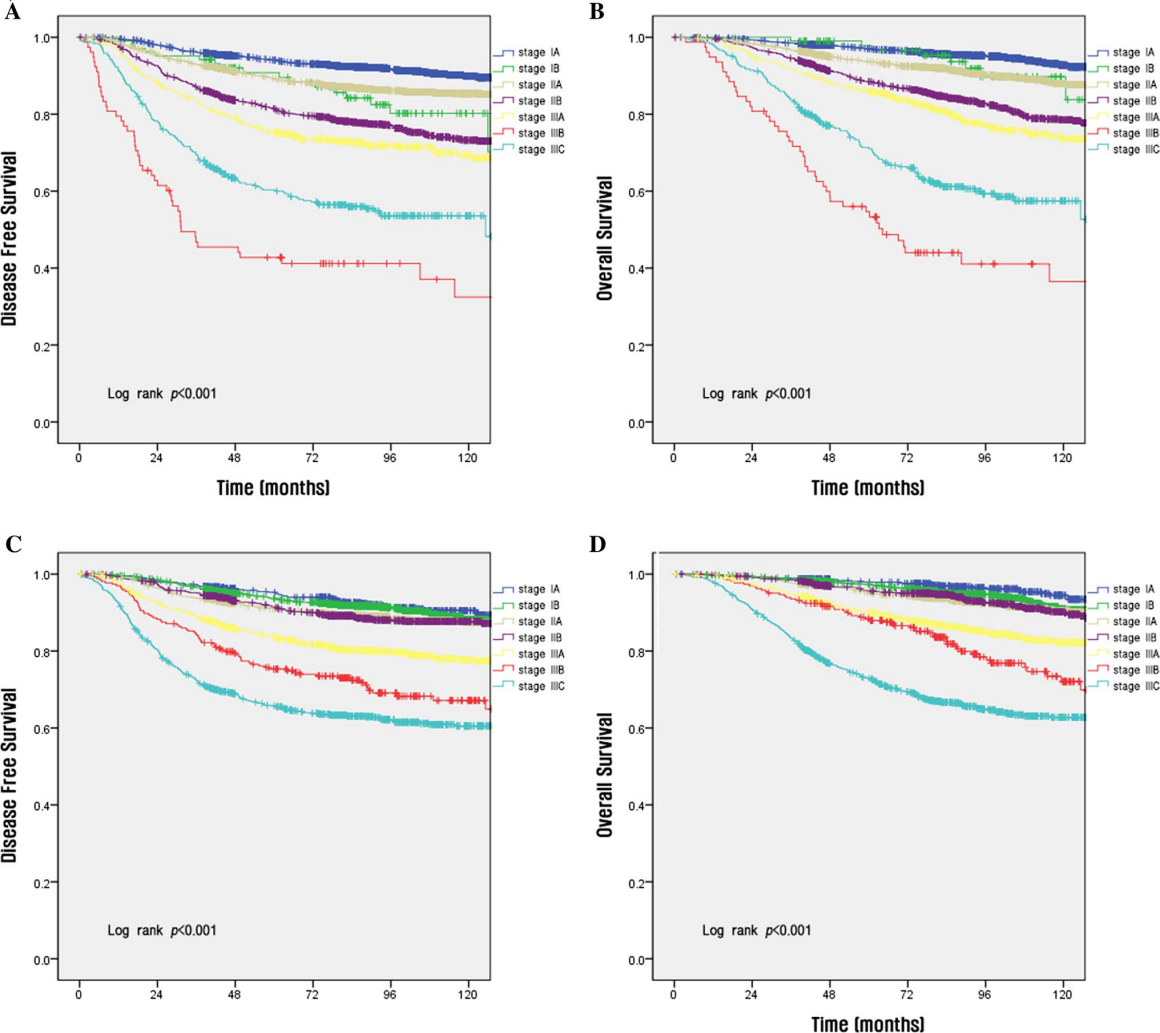
Disease-free survival (**a**, **c**) and overall survival (**b**, **d**) analyses within different disease stages using the 8th edition of the AJCC anatomic staging system (**a**, **b**) and prognostic staging system (**c**, **d**)

**Fig. 2:**
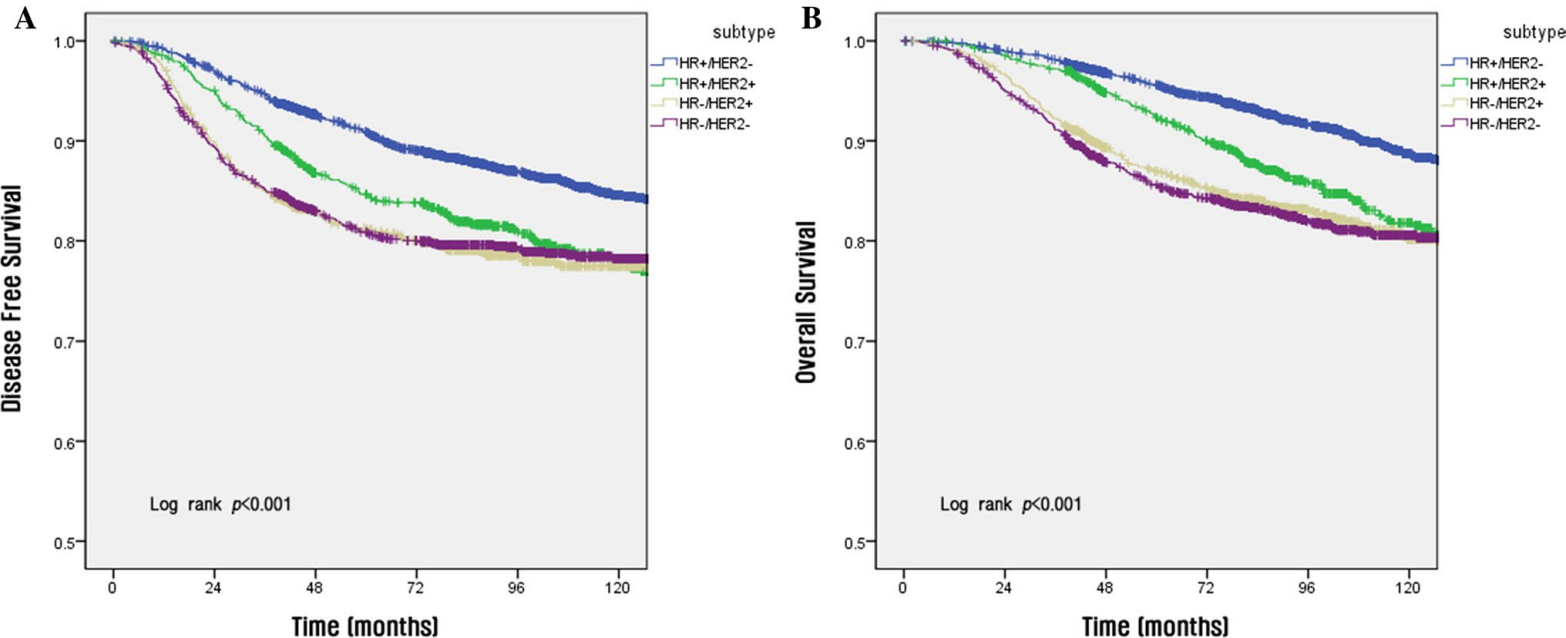
Disease-free survival (**a**) and overall survival (**b**) analyses according to tumor subtype

**Fig. 3:**
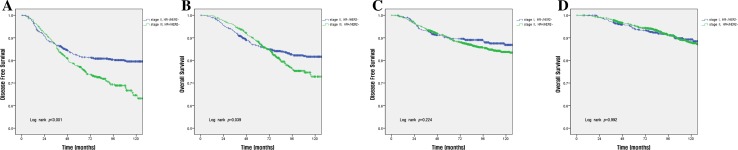
Disease-free survival (**a**, **c**) and overall survival (**b**, **d**) rates between patients with stage II (**a**, **b**) and I (**c**, **d**) HR(−)/HER2(−) tumors and those with stage III (**a**, **b**) and II (**c**, **d**) HR(+)/HER2(−) tumors using the anatomic staging system

**Fig. 4:**
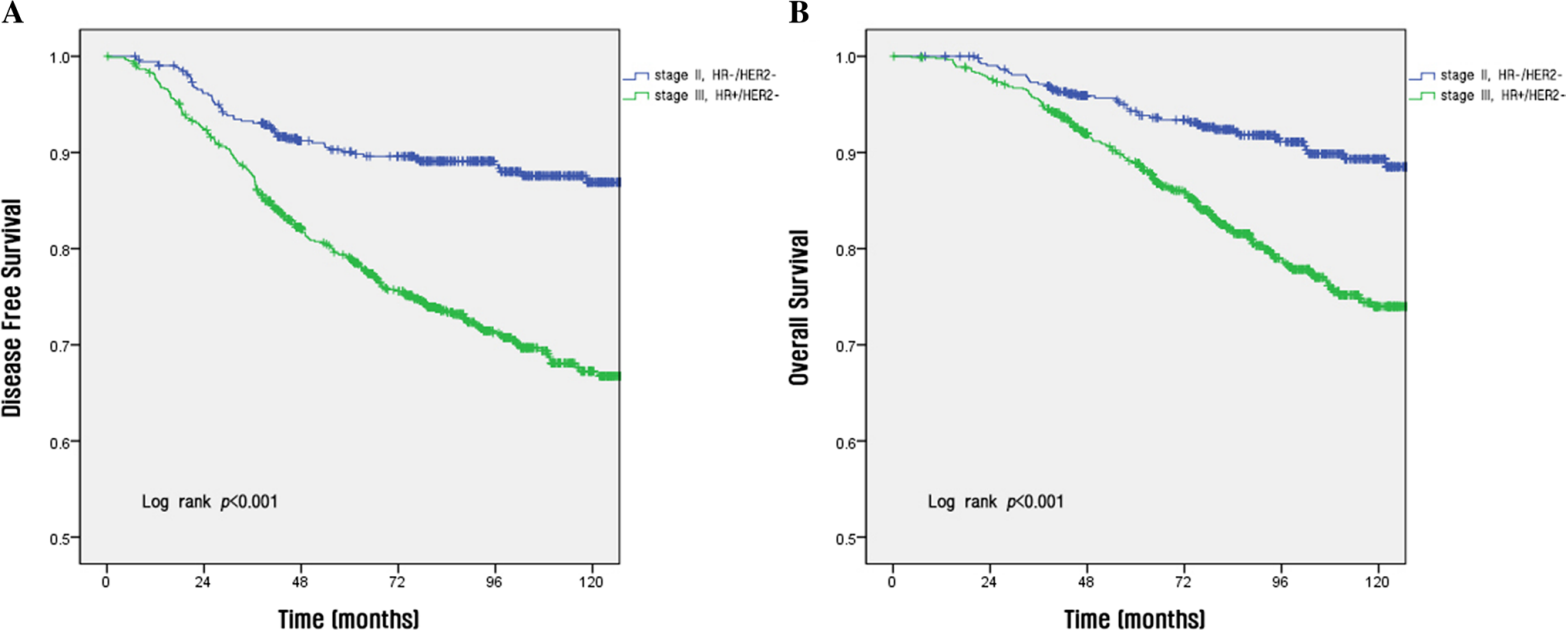
Disease-free survival (**a**) and overall survival (**b**) rates between patients with stage II HR(−)/HER2(−) tumors and those with stage III HR(+)/HER2(−) tumors using the prognostic staging system

The survival rate according to each stage per the anatomic and prognostic staging systems is shown in Fig. 1. Using the anatomic staging system, the 10-year DFS rates for patients with stage IB disease (80.2%) was lower than that of patients with stage IIA disease (85.4%) (a, b in Fig. 1). In contrast, when the prognostic staging system was used, the survival rate was found to decrease with increasing disease stage (c and d in Fig. 1). The 5-year OS by subtype was as follows: HR(+)/HER2(−), 90.9%; HR(+)/HER2(+), 84.7%; HR(−)/HER2(+), 81.1%; and HR(−)/HER2(−), 80.9% (Fig. 2). Using the anatomic staging system, the 5-year OS of patients with stage III HR(+)/HER2(−) disease was found to be better than that of patients with stage II HR(−)/HER2(−) disease (88.3 vs. 86.5%). The survival rate of patients with stage II HR(−)/HER2(−) disease was higher than that of patients with stage III HR(+)/HER2(−) disease in the early period after surgery, but crosses the survival rates at about 36 months after surgery in DFS and about 68 months in OS (a, b in Fig. 3). Survival rates of patients with HR(−)/HER2(−) of stage I (5-year OS, 94.3%) and HR(+)/HER2(−) of stage II (95.5%) also show a overlap at about 60 months in DFS and about 96 months in OS, respectively (c, d in Fig. 3). On the other hand, using the prognostic staging system, both the 5 and 10-year DFS and OS of patients with stage II HR(−)/HER2(−) disease (5-year DFS, 90.1%; 5-year OS 94.3%) were higher than those of patients with stage III HR(+)/HER2(−) disease (5-year DFS, 79.1%; 5-year OS, 88.9%) (a, b in Fig. 4). The survival rates of patients with stage I HR(−)/HER2(−) disease and those with stage II HR(+)/HER2(−) disease were not comparable, because no cases of stage I HR(−)/HER2(−) disease were identified using the prognostic staging system.

The proportion of patients assigned the same stage using both the anatomic and prognostic staging systems was lower than 50%, except for patients with stage III disease (91.8%) (italic values in Table 4). The proportion of patients with stage IA disease using the anatomic staging system but diagnosed with stage IB disease using the prognostic staging system was 50.7%. The rate of changes from stage IB disease using the anatomic staging system to stage IIA disease using the prognostic staging system was 32.6%. In particular, there were several cases of changes from stage IIB disease using the anatomic staging system to stage III disease (changes to stage IIIA disease, 22.8%; IIIB, 6.6%, and IIIC, 24.2%) using the prognostic staging system. This is because, among patients classified with an anonymous prognostic stage, the highest proportion of patients were diagnosed with stage IIB disease using the anatomic staging system (Table 4). The DFS and OS determined using the anatomic staging system and the prognostic staging system significantly increased with lower disease stage (Table 5). ER status, PR status, HER2 status, and tumor grade were added during the migration from the anatomic staging system to the prognostic staging system. However, anonymous cases were identified during in this process (Table 6).

**Table 4:** Changes in disease stages from anatomic stage groups to prognostic stage groups (*N* = 7458)

		AJCC8								
		IA	IB	IIA	II	IIIA	IIIB	IIIC	Anonymous	Total
AJCC7	IA	*647* *(22.5%)*	1455 (50.7%)	714 (24.9%)	0	0	0	0	54 (1.9%)	2870 (38.5)
	IB	20 (19.2%)	*48* *(46.2%)*	34 (32.6%)	1 (1.0%)	0	0	0	1 (1.0%)	104 (1.4%)
	IIA	0	193 (8.9%)	*276* *(12.7%)*	970 (44.7%)	732 (33.7%)	0	0	0	2171 (29.1%)
	IIB	0	68 (5.6%)	4 (0.3%)	*50* *(4.1%)*	278 (22.8%)	80 (6.6%)	296 (24.3%)	441 (36.3%)	1217 (16.3%)
	IIIA	0	33 (5.0%)	1 (0.2%)	22 (3.4%)	*214* *(32.7%)*	150 (22.9%)	233 (35.6%)	1 (0.2%)	654 (8.8%)
	IIIB	0	0	0	0	0	*4* *(5.%)*	74 (94.9%)	0	78 (1.0%)
	IIIC	0	0	0	0	0	30 (8.2%)	*334* *(91.8%)*	0	364 (4.9%)
	Total	667 (8.9%)	1797 (24.1%)	1029 (13.8%)	1043 (14.0%)	1224 (16.4%)	264 (3.5%)	937 (12.6%)	497 (6.7%)	7458

**Table 5:** Comparison of DFS and OS using the 8th edition of AJCC anatomic and prognostic staging system of luminal A breast cancer (*N* = 7458)

Stage	Anatomic stage groups					Prognostic stage groups				
	*n*	5-year DFS (%)*	p**	5-year OS (%)*	p**	n	5-year DFS (%)*	p**	5-year OS (%)*	p**
			< 0.001		< 0.001			< 0.001		< 0.001
I	2974	94.0		97.0		2464	94.1		97.5	
II	3388	86.4		91.8		2072	91.2		95.4	
III	1096	68.1		78.9		2425	75.9		83.2	

*DFS* disease-free survival, *OS* overall survival

*DFS and OS are analyzed by Kaplan–Meier survival analysis

**Log-rank test

**Table 6:** Anonymous cases

Anonymous cases	*T*	*N*	*M*	HG	ER	PgR	HER2	Number	%
1	1	0	0	2	(−)	(+)	(−)	55	0.7
2	2	1	0	2	(+)	(+)	(−)	253	3.4
3	2	1	0	2	(+)	(−)	(+)	28	0.4
4	2	1	0	2	(−)	(+)	(+)	14	0.2
5	2	1	0	2	(−)	(−)	(+)	45	0.6
6	2	1	0	3	(+)	(+)	(+)	44	0.6
7	2	1	0	3	(+)	(−)	(+)	20	0.3
8	2	1	0	3	(−)	(+)	(+)	11	0.1
9	3	0	0	1	(+)	(+)	(−)	2	0
10	3	0	0	2	(+)	(+)	(+)	3	0
11	3	0	0	2	(+)	(+)	(−)	13	0.2
12	3	0	0	2	(+)	(−)	(+)	2	0
13	3	0	0	2	(−)	(+)	(+)	1	0
14	3	0	0	2	(−)	(−)	(+)	3	0
15	3	0	0	3	(+)	(+)	(+)	1	0
16	3	0	0	3	(+)	(−)	(+)	2	0
Total								497	6.7

## Discussion

It has been 40 years since the first edition of the AJCC staging manual was published in 1977 [10]. This staging system has become the most effective cancer classification and prognosis evaluation system globally. The TNM scoring system—tumor size, lymph nodes affected, and metastasis—has traditionally been used for cancer staging [9], and thus, for evaluating the tumor burden. The TNM staging system has been revised several times and has become the most widely used and authoritative cancer staging system worldwide. However, because breast cancer is so heterogeneous, many researchers found that traditional anatomic staging alone cannot accurately predict prognosis and that pathological features rather than anatomical information reflect the essential characteristics of breast cancer more accurately [11].

Owing to the continual growth in cancer research, tumor subtype and recommendations for treatment of breast cancer have been provided at the 2011 St. Gallen international expert consensus statement. Clinicians around the world now recognize these guidelines as the best treatment approach for primary breast cancer [12]. The panel of this symposium provided a standard treatment protocol for breast cancer following the subtype classification [4]. Breast cancer is divided into luminal A, luminal B, normal-like, HER-2, and basal-like. Based on pathological information, such as histologic type, grade, ER status, PR status, HER-2 status, Ki-67 index, tumor size, and axillary lymph node status, we routinely select a particular treatment for a patient with early breast cancer [13]. Forty years since the St. Gallen conference, it is clear that the focus has shifted from anatomy to biology.

The 8th edition of the TNM staging system includes anatomic stage groups as well as prognostic stage groups, which incorporate biomarker testing that yields improved prognostic discrimination when compared with anatomic staging alone. Histological grade and ER, PR, and HER-2 status are incorporated to generate prognostic stage groups. The anatomic staging system overlooks subtype; therefore, survival rates among patients with lower disease stage can be lower than those of patients with higher disease stage. Therefore, a more accurate tool for prediction of prognosis is needed.

In our retrospective study, we analyzed the clinical significance of the prognostic staging system proposed in the 8th edition of the AJCC cancer staging system for breast cancer. Previous studies reported that ER(+) and/or PR(+) and HER-2(−) tumors comprised the most common subtype, followed by ER(+) and/or PR(+) and HER-2(+), triple negative, and HER-2 over-expressed tumors [8, 14, 15]. The overall distribution of subtype in our study was similar. The ER(+) and/or PR(+) and HER-2(−) subtype was the most common (52.9%), followed by triple negative (20.5%), HER-2 over-expressed (13.6%), and ER(+) and/or PR(+) and HER-2(+) (13.0%).

The subtype at stage I in the anatomic stage is the order HR(+)/HER2(−) (58.5%), HR(−)/HER2(−) (17.6%), and they were similar in stage II and III. By the way, in the prognostic stage, HR(+)/HER2(−) is the most common (67.3%), but no HR(−)/HER2(−) at stage I. In addition, the rate of HR(−)/HER2(−) in stage III was 22.5% in the anatomic stage, but increases to 41.5% in the prognostic stage (Table 3). Cases that could not be staged, anonymous cases, accounted for 6.7% of all patients and were classified into 16 groups (Table 6).

The classification of OS in the anatomic staging system was not adequate. Kim et al. reported that the survival rate of the IIIB group was lower than that of the IIIC group. A joint analysis of 9640 patients with invasive breast cancer showed that those with stage IIIB disease demonstrated a significantly worse DFS [hazard ratio, HR 10.4; 95% confidence interval (CI) 6.9–15.7] that those with stage IIIC disease (HR 7.2; 95% CI 5.9–8.7) [16]. Woodward et al. yielded similar results, showing a 15-year OS of 18% in stage IIIB disease patients and 28% in stage IIIC disease patients [17].

In our study, when cases were classified according to the anatomic stage, the 5-year OS and DFS rates were 54.7 and 42.8% for stage IIIB disease and 71.0 and 60.4% for stage IIIC disease. However, per the prognostic staging system, the 5-year OS and DFS rates for stage IIIB and IIIC disease were 88.4 and 75.3% and 72.9 and 65.8%, respectively. Moreover, the survival rate was found to decrease with increasing disease stage (c, d in Fig. 1). Therefore, the survival rate determined using the prognostic stage was superior to that achieved using the anatomic stage.

Furthermore, using the prognostic stage, the classification of survival according to subtypes by disease stage was clearer than that observed using the anatomic stage. In our study, breast cancer patients were staged according to anatomical staging, and in some stages or subtypes, the survival rate of patients with lower stage disease was lower than that of patients with higher stage disease. Per the anatomic stage, the 10-year DFS rates for those with stage IB disease (80.2%) was lower than those for patients with stage IIA disease (85.4%) (a, b in Fig. 1). Moreover, the 5-year OS rates of patients with stage II HR(+)/HER2(−) disease was superior to those of patients with stage I HR(−)/HER2(−) disease (95.5 vs. 94.3%; a, b in Fig. 3). Also, the 5-year OS rate of patients with stage III HR(+)/HER2(−) disease was better than those of patients with stage II HR(−)/HER2(−) disease (88.3 vs. 86.5%). However, per the prognostic stage, both the 5 and 10-year DFS and OS of patients with stage II HR(−)/HER2(−) disease (5-year DFS, 90.1%; 5-year OS 94.3%) were higher than those of patients with stage III HR(+)/HER2(−) disease (5-year DFS, 79.1%; 5-year OS, 88.9%) (a, b in Fig. 4).

Discrimination between each stage is clearer when the subtypes are classified using the prognostic stage rather than subtype classification using the anatomic stage. The DFS rate of patients with stage II HR(+)/HER2(−) disease and those with stage I HR(−)/HER2(−) disease overlap at 60 months, and that of patients with stage III HR(+)/HER2(−) disease and stage II HR(−)/HER2(−) disease overlap at 36 months per the anatomic stage, but these value are well separated when the prognostic stage was applied (Figs. 3, 4).

Survival curves generated using the anatomic stage intersect because the survival rate of patients with luminal type breast cancer is higher than that of patients with triple-negative cancer during the early period after surgery, but the recurrence rate is higher in patients with luminal type cancer as the postoperative period progresses. In the 1900s, Saphner et al. showed that the risk of recurrence in ER-negative patients was higher during the first 5 years after surgery, but decreases significantly over time. Also, the risk of recurrence in patients with ER-positive disease remained relatively constant. There was an overlap of recurrence risk among ER-negative and ER-positive patients between 3 and 4 years after surgery, and the risk of recurrence was higher in ER-positive patients 5 years after surgery. This study has contributed greatly to the understanding of recurrence patterns of invasive breast cancer and has been extensively cited in many other clinical studies [18]. Also, Park et al. demonstrated a variety of HRs according to the molecular diagnoses of breast cancer in a cohort of patients diagnosed between 1999 and 2005. The HR of HER2 positive and triple-negative breast cancer was highest at about 1 year after the diagnosis of breast cancer [19].

There are several limitations in our study. First, the use of Oncotype Dx in our analysis Korea was limited because the assay was not approved by the government at that time. Our single-center, retrospective study involved a relatively small number of patients. In order to achieve greater clinical value, a multi-center retrospective study is needed. In our group of patients, 259 (2.3%) were missing ER-related information, 301 (2.7%) were missing PR-related information, and 493 (4.4%) were missing HER2-related information. Approximately 2490 (22.4%) cases were missing grade-related information. Moreover, the cutoff for ER and PR positivity used in the present study was > 10%. However, the American Society of Clinical Oncology/College of American Pathologists recommended a cutoff of 1% of tumor cells positive for ER/PR for a specimen to be considered positive in 2010 [20]. Owing to evidence indicating that patients with HER2-positive tumors treated with trastuzumab have superior survival rates, since 2009, the Korean health insurance has covered trastuzumab; however, this group did not receive trastuzumab [2].

## Conclusions

In conclusion, the 8th edition of the AJCC prognostic staging system is an important supplement to the current breast cancer staging system. We conclude that staging of cancer on the basis of the prognostic stage is a more accurate system for prediction of prognosis and classification of survival rate for breast cancer than staging of cancer on the basis of anatomic stage.
